# Miniaturization and Model-Integration of the Optical Measurement System for Temperature-Sensitive Paint Investigations

**DOI:** 10.3390/s23167075

**Published:** 2023-08-10

**Authors:** Jonathan Lemarechal, Benjamin Daniel Dimond, Hans Peter Barth, Michael Hilfer, Christian Klein

**Affiliations:** Institute of Aerodynamics and Flow Technology, German Aerospace Center (DLR), Bunsenstrasse 10, 37073 Göttingen, Germany; benjamin.dimond@dlr.de (B.D.D.); peter.barth@dlr.de (H.P.B.); michael.hilfer@dlr.de (M.H.); christian.klein@dlr.de (C.K.)

**Keywords:** temperature-sensitive paint (TSP), camera miniaturization, camera integration, cross-flow instability, *SPECTRA-A*

## Abstract

The temperature-sensitive paint (TSP) method, an optical measurement technique, is used for qualitative skin friction visualizations in a wide variety of aerodynamic applications. One such application is the visualization of the laminar–turbulent boundary-layer transition. Optical access to the surface of interest is mandatory for the measurement system, which consists of scientific cameras and LEDs. But the optical access to the area of interest is often impeded by the available windows of the wind tunnel and the wind tunnel model itself, reducing the field of view and the spatial resolution. In some cases, it is of interest to increase the flexibility of the installation of the optical measurement system by reducing its physical dimensions and placing the installation inside the plenum. The DLR Swept flat PlatE Cross-flow TRAnsition (*SPECTRA-A*) configuration was selected to investigate the influence of two-dimensional steps on the cross-flow-induced boundary layer transition by means of TSP, as part of the EU project Clean Sky 2. The *SPECTRA-A* configuration consists of two main elements: a flat plate and a displacement body mounted within a very close distance of each other, creating a narrow gap between the two elements. The surface of interest is the area on the flat plate facing the displacement body. The narrow gap limits the utilization of an external camera setup due to poor optical access. A new optical setup consisting of four miniature CMOS machine-vision cameras and five miniature high-power LEDs was integrated into the displacement body. The characteristics of the camera system were analyzed in laboratory tests, establishing that the miniature CMOS machine-vision cameras are suitable for qualitative TSP skin friction visualizations. This was confirmed by successfully measuring the laminar–turbulent boundary-layer transition on the *SPECTRA-A* configuration. The integrated TSP system is capable of resolving even small variations of the transition location caused by changing the amplitude of the stationary cross-flow instability. The quality of the TSP visualization with the integrated optical system allows for the measurement of the transition location and the wavelength of the stationary cross-flow instability. Overall, a cost-effective TSP visualization system with small space requirements was developed and tested for future applications in wind tunnel models, model support, or side walls of wind tunnels.

## 1. Introduction

The temperature-sensitive paint (TSP) method is a non-intrusive, reversible, optical surface-temperature measurement technique. It is applied in wind tunnels and water facilities for surface-temperature measurements [1,2], heat-flux measurements [3,4], skin friction measurements [5], and qualitative skin friction measurements [6], i.e., flow visualizations. The TSP measurement system for aerodynamic or hydrodynamic applications is composed of the TSP coating, consisting of temperature-sensitive luminophores embedded in a suitable binder, and the optical measurement system, i.e., an illumination and photosensitive device [7]. Typically, the TSP is illuminated by LED or a laser, and CCD or CMOS cameras are used as photosensitive devices. TSP data can be acquired via the intensity-based method [7], lifetime-based method [7], and frequency-domain fluorescence lifetime imaging (FLIM) [7]. The different methods have significantly different capabilities and requirements concerning the accuracy of the acquisition system, e.g., the sensitivity, linearity, and accuracy of the timing of the image acquisition, as well as illumination power and illumination characteristics. Consequently, scientific-grade equipment is commonly used, which is typically fragile, costly, and large. Thus, it needs to be installed in a safe way outside of the wind tunnel’s test section, often far away from the surface of interest.

**Figure 1:**
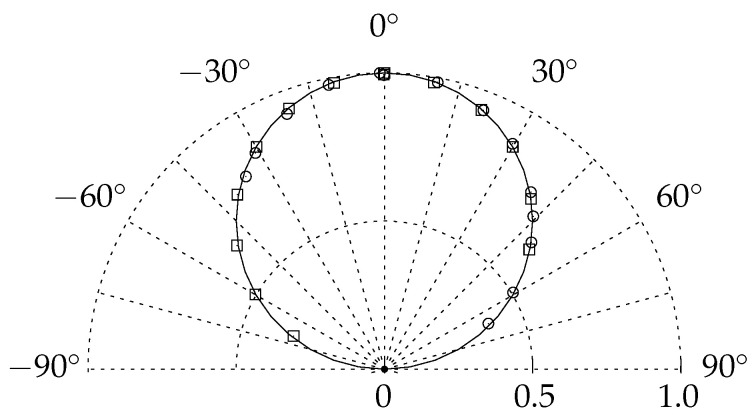
Angular dependency of the excitation (□) and emission (°) of Eu-based TSP in comparison to Lambert’s cosine law (**—**). [7].

Restrictions of the optical access do not only affect the spatial resolution, but the measurable intensity emission is influenced by the distance and the angle between the TSP surface and the camera and LED. Preliminary tests within the scope of this publication showed that the dependency of TSP excitation and emission is in good agreement with Lambert’s cosine law (Figure 1). The investigation also showed that the results are not significantly influenced by the surface roughness, when surface quality requirements for aerodynamic application are complied. For experimental setups with an acute angle between surface and optical axis, the angular dependency of the excitation and emission of TSP should be taken into account during the design of the experiments. The increased area, which is recorded by a single pixel for smaller angles between the camera view axis and model surface, compensates for the decrease of emitted light. However, a small angle between the illumination axis and the model surface is not compensated. Examples of TSP measurements with severe limitations of the optical access due to the construction of the test section are the cryogenic Ludwieg tube (KRG) [8] and the Pilot European Transonic Windtunnel (PETW) [9].

The experimental configuration, known as *SPECTRA-A* (Swept flat PlatE Cross-flow TRAnsition), a wind tunnel configuration [10], serves as one of the technology demonstrators for the Clean Sky 2 initiative [11]. The model is designed for research in cross-flow-dominated transition scenarios [12] and is based on the cross-flow experiment set up by Bippes et al. [13,14,15,16,17]. In the Clean Sky 2 project, the *SPECTRA-A* configuration is utilized to investigate the aerodynamics of cross-flow-dominated laminar–turbulent boundary-layer transition, specifically when influenced by two-dimensional surface irregularities. For detailed information on the effect of steps on the cross-flow-dominated laminar–turbulent transition, see [18,19,20,21].

For this investigation, the intensity-based TSP method is used for transition measurement. The design of the *SPECTRA-A* configuration considerably limits the direct view of the surface of the investigation. The investigation is conducted in the one-meter wind tunnel Göttingen (1MG) of the German Aerospace Center (DLR) Göttingen, Institute of Aerodynamics and Flow Technology. If standard scientific cameras and high-power LEDs were used for the measurements, the nearest practically possible installation location would be located just outside of the open test section, 2.5 m downstream of the flat plate’s trailing edge. The required amount of light sources for excitation would be large due to the disadvantageous illumination angle (Figure 1). To achieve high-quality TSP visualizations with a high spatial resolution, it is necessary to integrate the optical measurement system into the displacement body, which blocks the view. Due to the dimension of the displacement body a miniaturization of the optical measurement system is required.

Rapid developments in the field of cameras for machine-vision applications and high-power LEDs for UV curing and disinfection have led to an expanding array of affordable cameras and LEDs suitable for TSP applications. An initial application of a machine-vision camera for pressure-sensitive paint (PSP) calibration was demonstrated by Quinn et al. [22]. For qualitative skin friction measurements, the intensity-based TSP method is predominantly applied. This method has less strict requirements concerning the quality of cameras and LEDs. Therefore, low-priced cameras can be chosen for integration in wind tunnel models or wind tunnel walls. Also, the flush integration of LEDs for homogenous, continuous illumination of models can be achieved with low-cost LEDs. Furthermore, measurements can be conducted where the loss of equipment cannot be excluded. Hence, the miniaturization of the optical measurement system using machine-vision cameras as well as a simple high-power LED setup for qualitative skin friction measurements are particularly suitable for qualitative TSP visualizations. The miniaturized optical measurement system benefits the field of view (FOV), spatial resolution, and illumination uniformity.

In this paper, we present the results of a laboratory test and a TSP calibration to evaluate a machine-vision camera’s suitability for performing time-averaged flow visualizations with TSP. The camera used in this paper is significantly smaller and costs less than scientific cameras and previously investigated machine-vision cameras [22]. Four of these cameras are integrated into the displacement body of the *SPECTRA-A* configuration together with five high-power LEDs for TSP illumination. The suitability of this miniaturized and integrated TSP setup for transition measurements is tested by investigating the influence of a backward-facing step on the transition location of stationary cross-flow-induced transition. The measurements are conducted for different flow velocities and a variation of the amplitude of the stationary cross-flow instability, which is altered by changing the artificially applied surface roughness near the leading edge.

The integration of the miniaturized optical measurement system for TSP data acquisition into the *SPECTRA-A* model enables fast measurement of the location of laminar–turbulent transition over a large portion of the model surface, thereby facilitating extensive parameter studies in the future. This work demonstrates for the first time that TSP flow visualizations of good quality are achieved with commercial low-cost miniature machine-vision cameras and commercial LED chips. In this paper, we first present the experimental methods in Section 2. Afterward, the suitability of the miniature camera for TSP flow visualizations and transition measurements is tested in laboratory tests (see Section 3) and applied to aerodynamic testing when integrated into the *SPECTRA-A* configuration; see Section 4. Finally, the results of these tests are discussed and summarized in Section 5 and Section 6, respectively.

## 2. Methods

### 2.1. 1-m Wind Tunnel Göttingen

The wind tunnel test was conducted in the one-meter wind tunnel Göttingen (1MG) of the German Aerospace Center (DLR) Göttingen, Institute of Aerodynamics and Flow Technology. It is a Göttingen-type low-speed wind tunnel. The turbulence level of the flow in the empty short test section is in the order of 0.2% at 20 m s^−1^ in the frequency range of 10 Hz to 10^4^ Hz [23]. Measurements with the long open test section and the *SPECTRA-B* configuration showed a similar turbulence level of 0.18% for typical flow conditions [24]. For the test, the long open test section with a length of 2.4 m is installed. The rectangular nozzle has a width of 1 m and a height of 0.74 m, which is modified with a triangular nozzle adapter in such a way as to achieve a constant chordwise distance of the nozzle exit to the leading edge of the flat plate [14]. The temperature of the flow is not conditioned by the facility.

### 2.2. SPECTRA-A Configuration

The *SPECTRA-A* configuration provides a pressure distribution and boundary-layer stability characteristics similar to the original cross-flow experiment set up by Bippes et al. [13,14,15,16,17], but with a larger chord length. The experiment was first reestablished with different pressure distributions and boundary-layer stability characteristics suitable for the investigation of a transition control technique called upstream flow deformation [25]. In that configuration, it is named *SPECTRA-B* [10,24]. For both *SPECTRA-A* and *SPECTRA-B*, the boundary layer under study is stable with respect to all typical primary instabilities in a swept configuration, except for cross-flow instabilities.

**Figure 2:**
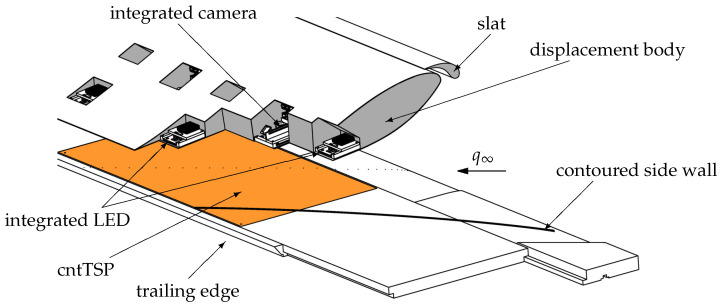
Isometric view of the *SPECTRA-A* configuration with breakouts showing some of the integrated LEDs and miniature cameras. The contoured side walls and nozzle adapter are not shown, except for the indicated shape and location of one side.

An isometric view of the *SPECTRA-A* configuration is shown in Figure 2. The configuration is installed with a sweep angle of $\phi_{\infty }=$ 45°. The area of interest for the investigation of the cross-flow instability is the upper side of the flat plate, which has a chord length of c= 600 mm. The displacement body arranged above the flat plate imprints a negative pressure gradient on the plate. A slat is installed in front of the displacement body to avoid flow separation on the displacement body’s upper side. The slat and displacement body are made of carbon-fiber-reinforced plastic (CFRP). The flat plate is made of aluminum and consists of three parts, i.e., a leading-edge module, the main module ($0.25\leq x_{c}/c\leq 0.96$), and a trailing-edge segment. This modular design provides the possibility to install forward-facing or backward-facing steps at $x_{c}/c=0.25$. The flat plate features 40 pressure taps arranged parallel to the main axis of the free stream velocity. Their spacing is mostly equidistant, except for a narrower spacing near the attachment line.

Contoured side walls and the nozzle adapter make it possible to approximate infinite swept wing flow in the experiment despite the configuration’s high blockage [24]. The contoured side walls are shaped like the streamline at the upper boundary-layer edge as derived from numerical calculations. The nozzle adapter elongates the wind tunnel’s nozzle to ensure parallelism between the nozzle exit and the leading edge of the flat plate [14].

In the presented work, the flat plate is set up with a backward-facing step that is 0.45 mm in height. The velocity of the 1MG $U_{\infty }$ varies between 25 and 35 m s^−1^. Due to the severe blockage of the test section by the *SPECTRA-A* configuration, the velocity measurement provided by the wind tunnel is not identical to the velocity $q_{\infty }$ of the infinite swept wing configuration (Figure 2). Therefore, the velocity $q_{\infty }$ has to be derived from a numerical solution, which is adjusted to match the experimental chordwise velocity distribution at the curved boundary-layer edge streamline, which is measured by two-component hot-wire anemometry.

The turbulence level of the 1MG leads to similar amplitudes of traveling and stationary cross-flow instabilities in the investigated boundary layer. In order to ensure that the laminar–turbulent transition is predominantly caused by stationary cross-flow instabilities and to improve the reproducibility, they are artificially excited with a spanwise-equidistant arrangement of discrete roughness elements (DREs) [26,27]. The DREs are installed by applying one, two, and three layers of rub-on dots (⌀ = 2.5 mm; height ∼ 10 μm), leading to a total height of the DRE of approximately 10, 20, and 30 μm, respectively. From an accompanying LST analysis, a stationary cross-flow instability mode was chosen, which showed it to be strongly amplified across the entire range of planned experimental conditions. In order to excite the chosen instability mode, the DRE is installed with a spanwise spacing of λ= 9.5 mm at $x_{c}/c=0.066$, which is close to the indifference location.

Two coordinate systems are used to present the TSP results in addition to the pixel-based image plane. Firstly, the plate-oriented coordinate system $x_{c},y_{c}$, which is aligned with the plate’s chordwise and spanwise directions, is used. Secondly, the vortex-oriented coordinate system $x_{c},y_{v}$ is aligned with the vortex axis of the stationary cross-flow vortices.

#### Model Integration of the TSP Measurement System

The integration of the TSP measurement system (i.e., the TSP coating, electrical heating, optical acquisition system, and illumination system) into the *SPECTRA-A* configuration is described hereafter.

The TSP coating and electrical heating based on carbon nanotubes (CNTs) [28] were successfully integrated into the wind tunnel model (the flat plate) without disturbing the model contour. The combination of TSP with the integrated CNT model heating will be called cntTSP, hereafter. The cntTSP is integrated into the flat plate via a 0.35 mm deep pocket of $\Delta x_{c}\times \Delta y_{c}$ = 400 × 800 mm^2^ in the main plate module. Initially, a thick layer of polyurethane (PUR) is applied with a spray gun into the pocket. After drying of the PUR coating, the copper electrodes are glued to the PUR surface and the CNT layer is applied with a spray gun. Afterward, a very thin white screen layer is applied with a spray gun to increase the luminous efficacy of the TSP. After curing, markers required for image registration are applied to the screen layer. Subsequently, the active layer of the TSP is applied by a spray gun. Finally, the surface quality, the intersection to the metal surface, and the shape of the pressure taps are improved. For the *SPECTRA-A* configuration, a Europium-based TSP with high temperature sensitivity (3.6% K^−1^ at 30 °C) and negligible pressure sensitivity is chosen [29]. The applied TSP is excited in the UV range and emits light at approximately 615 nm. The cntTSP is applied in a rectangular shape, despite the limitations of the contoured side walls (Figure 2), to avoid temperature variations due to variations in the electric field in the CNT. Moreover, 17 pressure taps are located in the area of the cntTSP. At the location of the pressure tap orifice, a circular area is not coated with CNT in order to avoid any electrical contact with the aluminum substrate. The overall resistance of the CNT layer is $R_{CNT}=$ 80 Ω.

The design of the *SPECTRA-A* configuration and the installation in the 1MG provide only limited optical access to the flat plate surface. As shown in Figure 2, the contoured side walls, the displacement body (located only z= 120 mm above the flat plate at $x_{c}/c=0.5$), and the nozzle adapter block most of the optical access. Hence, the TSP optical measurement system, i.e., cameras, and LEDs, are completely integrated into the displacement body of the *SPECTRA-A* configuration; see Figure 2.

The Vision Components VC MIPI IMX296 camera, which is based on the Sony IMX296 CMOS sensor, was chosen for model integration. This monochromatic sensor provides 1.56 megapixels, a 10-bit dynamic, and a global shutter. The camera can operate up to 60.3 frames per second at full resolution. The space required to install the IMX296 camera is small, with an overall size of the camera sensor measuring 22×23.5×10 mm^3^ plus the lens. To operate the sensor, it needs to be connected to a small single-board computer, e.g., a Raspberry Pi 4, via flat flexible printed circuit cables.

**Figure 3:**
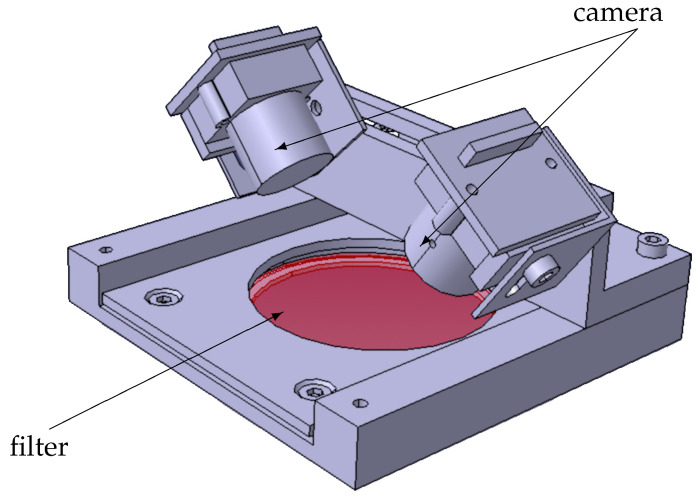
Isometric view of the dual camera setup.

In order to provide tailored optical access and space for the installation of the cameras and single-board computers, the CFRP displacement body is cut open from the top side. The optical access to the flat plate is enabled by holes with a diameter of 50 mm on the lower side of the displacement body. The location of these openings was previously determined in a laboratory test setup. The short distance between the displacement body and the flat plate requires four cameras to record the entire cntTSP area of $400\times 800\;{\mathrm{mm}}^{2}$. The number of holes to be cut into the displacement body is minimized by arranging two cameras with one shared optical access, as shown in Figure 3. The metal bracket stiffens the displacement body, fixes an optical filter, and provides support for the cameras. The lower side of the displacement body is closed by N-BK7 glass with a 50 mm diameter, 1 mm thickness, and an anti-reflection coating for the wavelength range of 400 nm to 700 nm. Due to the flat underside of the displacement body, the influence of the planar glass plate on the pressure distribution on the flat plate is expected to be negligible.

As a result of a laboratory pretest, lenses were selected with a focal length of 3 mm and a fixed aperture of f/5.6. Due to the short focal length and the inclined viewing angle through the filter (Figure 3), a wide range of angles under which the emitted light passes through the filter occurs. This is why colored glass high-pass filters with a cut-off wavelength of 550 nm are installed to avoid intensity loss caused by filter leakage. The optical setup provides images with an average spatial resolution between 0.2 mm/px and 0.4 mm/px.

The LEDs are also installed in specifically installed cavities in the displacement body, which are accessible from the top side. In the laboratory pretest, the numbers and locations of the LEDs were optimized. The installed setup consists of five Luminus UV CBT-120-UV LEDs. This LED type has an emission peak at 385 nm and is, therefore, well-suited for the applied TSP. For the LEDs, low-pass filters with a cut-off wavelength of 550 nm are installed.

The LEDs and small single-board computer of the optical measurement system are powered by power supplies located outside of the configuration. In this way, the space required for the LEDs can be greatly reduced. The power cables are routed through the spanwise ends of the displacement body, alongside LAN cables, to connect the small single-board computers with a data server. The cooling of the entire system, i.e., cameras, single-board computers, and LEDs is provided by pressurized air, which is guided into the displacement body through tubes and escapes at both spanwise ends.

**Figure 4:**
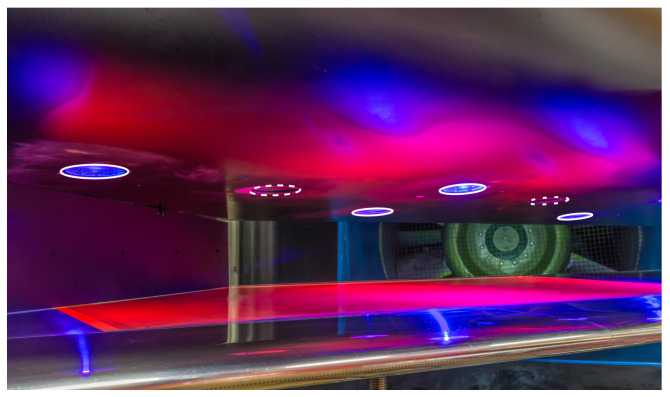
Photo of the *SPECTRA-A* configuration with integrated TSP in the 1MG. The view is oriented parallel to the main axis of the free stream velocity. The optical accesses for the LEDs (solid white lines) and cameras (dashed white lines) are marked. Courtesy of J. Agcos.

A photograph of the space between the flat plate and the displacement body of the *SPECTRA-A* configuration with integrated and switched-on LEDs is shown in Figure 4. The circular windows in the displacement body provide optical access for LEDs (illuminated in blue), and the camera windows (not illuminated) can be distinguished. Furthermore, the emitted red light of the TSP is visible. Because of inhomogeneities of the illumination light, some regions provide less emitted light and cause darker regions in the TSP area.

### 2.3. TSP Method

Temperature measurements with the TSP method rely on the temperature-dependent light emissions from luminophores. Initially, these luminophores are excited by light. Subsequently, they return to the ground state mainly by one of two processes: a radiationless de-excitation (thermal quenching) or the emission of light. Thermal quenching becomes more likely with increasing temperature, thus reducing the de-excitation by the emission of light and therefore the overall emission, resulting in temperature-dependent emission. An in-depth description of the photophysics of TSP is given by Liu et al. [7]. The luminophores are applied to the surface of wind tunnel models by spray painting a mixture of the luminophores and a transparent binder, i.e., a clear coat. Thus, the TSP method provides a high density of temperature probes, which is defined by the density of luminophores in the binder but limited by the spatial resolution of the camera and the quality of the optical system.

Thermographic measurement techniques, such as TSP or infrared thermography, require artificial heat flux between the model surface and fluid at low flow speeds to perform qualitative skin friction measurements [6]. When the heat flux is applied, the local temperature difference between the model surface and the flow is determined by the artificial heat flux and the local skin friction. The resulting visualization image can be used to derive the location of the laminar–turbulent transition by means of the largest temperature gradient in the flow direction [6,30]. For the investigation of the laminar–turbulent transition with dominating stationary cross-flow instability of the boundary layer, the spanwise wavelength of the dominant mode can be detected in the non-linear growth regime [31].

The measurement sequence for the *SPECTRA-A* configuration in the 1MG to acquire TSP data for a range of velocities consists of six phases: dark, reference, heating-up, run, cool-down, and post. First, the wind tunnel is set to the lowest velocity of the velocity range. Ten images are acquired each in the dark and reference phase. The dark images are acquired without excitation light and heating to acquire the background light. Also, the reference images are acquired without heating but with excitation light to acquire the intensity distribution for the temperature equilibrium of the fluid and the model. During the heating-up phase, no images are recorded, but an electric current is applied to the CNT with an electrical power of 196 W. After 300 s of heating the cntTSP and the flat plate, the surface temperature is increased by approximately 3.5 K in the laminar region. This temperature difference corresponds to a temperature ratio of $T_{surface}/T_{fluid}\approx 1.01$ between the surface and flow. In a previous investigation of cross-flow instability-induced transition with TSP [31], the influence of over-temperature on the transition location was negligible in the tested range of up to $T_{surface}/T_{fluid}=1.08$. Subsequently, the run phase started. During the run phase, ten images were acquired with excitation light and the heating still on for each velocity. Up to six velocities were acquired in one sequence. After the cool-down phase of approximately 300 s, the last ten images were acquired in the post-phase again without artificial heat flux.

#### Evaluation

**Figure 5:**
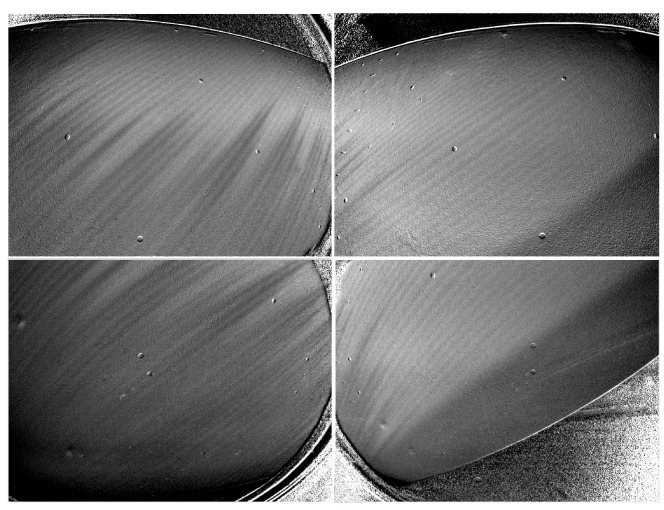
Perspective TSP result of each camera in the image plane for $q_{\infty }=$30 m/s.

The DLR in-house software *nToPas* [32] was used to perform the TSP data reduction in the image space and the projection onto a structured grid. The first step of the evaluation is averaging the images of each camera for each phase individually. Subsequently, the dark images are subtracted from both the reference and run images. Dividing the average reference image by the average run image of each camera provides the perspective result images [32]. Figure 5 shows the TSP results of each camera for one data point. In the results, larger skin friction, e.g., turbulent flow or footprints of stationary cross-flow vortices, can be distinguished from regions with lower skin friction, i.e., laminar flow, by darker and brighter shades of gray, respectively. In addition to the flow structures, the applied markers remain visible as black-and-white colored dots. This is caused by model deformation, which is dominated by the deformation of the displacement body. The applied image registration—to align the reference images to the run images for different velocities—provides only a reduction in the influence of the deformation. More markers would have been required to further reduce the influence of the model deformation.

**Figure 6:**
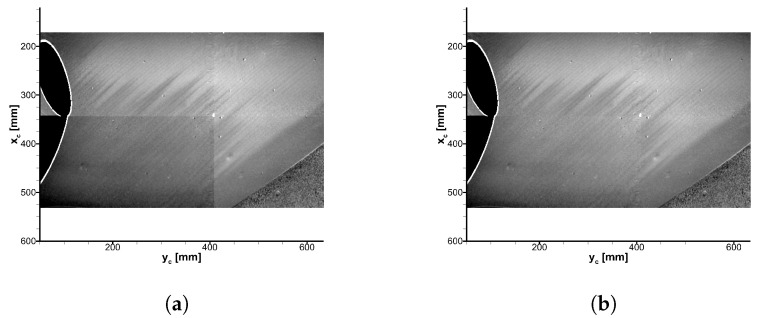
TSP results mapped onto a structured grid in the plate-oriented coordinate system. The flow direction is from the top right to bottom left. (**a**) Without the adjusted intensity ratio level. (**b**) With the adjusted intensity ratio level.

The images are projected onto a structured grid with 0.1 × 0.1 mm^2^ spacing by means of the model markers. Since the locations of the markers are determined in the reference image, and their respective locations are known in physical space, the camera projection matrix can be solved using the least square method. Details on the implementation of the extrinsic and intrinsic parameters and the projection method are provided by [8]. In Figure 6a, the projected and merged results of the data presented in Figure 5 are shown.

Taking a closer look at Figure 6a, it is apparent that the average ratio level and the noise level of the camera capturing the bottom left corner are different from the other cameras. This variation occurred randomly and is not limited to one camera; see also Section 3.3. The step in the intensity ratio at the junction of the camera views complicates the gradient-based evaluation of the transition location. Therefore, the intensity ratio is adjusted by evaluating the intensity ratio in the overlap regions for each camera pair. Consequently, an offset correction is applied to the intensity ratio to minimize the differences followed by a Gaussian filter on the merged result. The result with a successfully improved intensity-ratio distribution is presented in Figure 6b.

When examining the TSP results of the entire investigated velocity range, it becomes apparent that an area with a locally lower surface temperature appears. This area is located close to the trailing edge of the cntTSP and appears when the CNT heating is operated for longer times, i.e., several run images are acquired consecutively. It is assumed that a locally different CNT layer thickness causes the reduced surface temperature. This surface temperature inhomogeneity is diminished by dividing the TSP result with a TSP result containing only temperature variations of the heating. This result is acquired by dividing a TSP image, which was acquired after a similar heat-up time without flow, by a reference image without heat flux.

The transition location is derived by determining the local minimum of the gradient of the intensity ratio in a streamwise direction $\partial (I_{ref}/I)/\partial x$ [6,30]. For the cross-flow-induced laminar–turbulent transition, it is necessary to conduct the evaluation in the vortex-oriented coordinate system $x_{c},y_{v}$ to avoid shadowing effects of the saw-tooth pattern in the plate-oriented coordinate system $x_{c}$, $y_{c}$. The vortex axis is derived from the footprint of the stationary cross-flow vortices in the TSP results of a predominantly laminar test case. This footprint is used to perform a shift of the spanwise $y_{c}$ location parallel to the leading edge, which results in the utilized vortex-oriented $y_{v}$ coordinate. The transition evaluation is limited to the rectangular shape in the $x_{c},y_{v}$ coordinate system, where the entire length of the FOV can be evaluated.

## 3. Qualification of the Miniaturized Camera for TSP Acquisition

In this section, the suitability of the chosen machine-vision camera, VC MIPI IMX296, for TSP applications is examined. Important characteristics of a camera used in TSP visualizations include a global shutter, a linear dependency between light incidence and the output signal, uniform pixel sensitivity, and a consistent signal under constant light incidence [7]. The available information regarding these criteria is sparse for commercially available machine-vision cameras. Therefore, four cameras of one camera type were used for the necessary laboratory tests. The IMX296 is compared to a scientific monochromatic camera (PCO.4000), which is used in a wide range of different TSP and PSP applications at DLR, in Table 1.

### 3.1. Linearity

**Figure 7:**
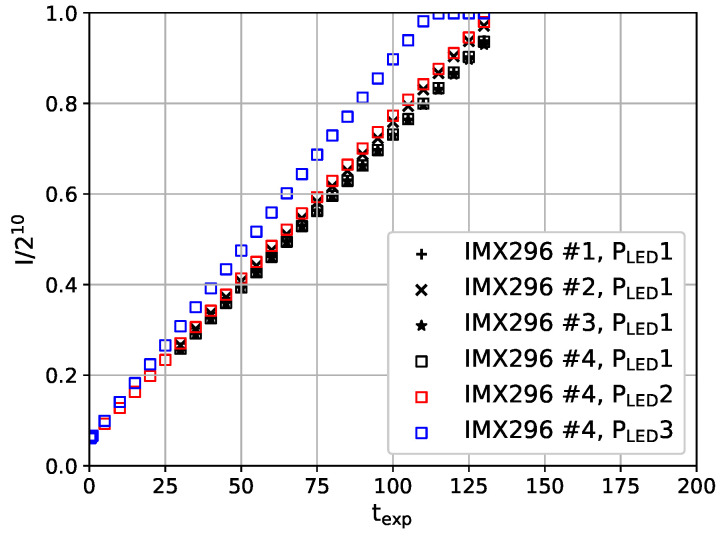
Dependency of the normalized intensity $I/2^{10}$ on the exposure time $t_{exp}$ for different IMX296 cameras and different brightness levels of the observed surface, i.e., different LED power settings $P_{LED}$. The individual cameras are distinguished by the provided number, e.g., #1.

The data evaluation of TSP measurements for flow visualization relies on the pixel-wise division of images, which were acquired at different surface temperatures, i.e., different intensity levels. For the investigation of the camera linearity, they were pointed at a white surface, which was illuminated by an LED. Images were acquired with an exposure time $t_{exp}$ that varied between 0.1 ms and 130 ms, which are the minimum and maximum possible exposure times of the camera. In the evaluation, a large area of the acquired image is averaged and the derived intensity is normalized by the maximum dynamic range of $2^{10}$. Figure 7 shows that all tested cameras provide the desired linear dependency of the measured intensity on the exposure time. Nevertheless, small variances are visible in Figure 7, which are most likely caused by minor differences in the LED power $P_{LED}$ and setup, which was designed to test the four cameras, one after another, with the same setup and LED power setting. When increasing the brightness of the white surface by increasing the LED power, the saturation of the sensor is reached before the maximum exposure time. The camera still shows linear behavior close to saturation (blue curve in Figure 7). Thus, the linearity characteristics of the camera are suitable for TSP visualizations.

### 3.2. Flat Field

**Figure 8:**
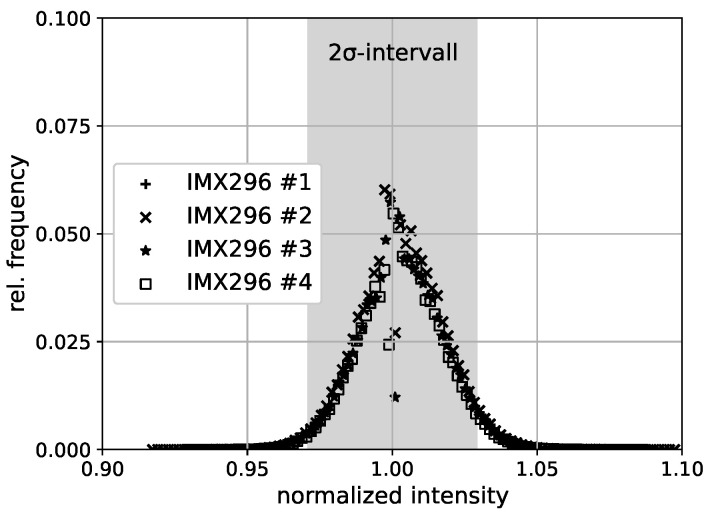
Histogram of normalized flat field images, acquired with the four investigated IMX296 cameras. The individual cameras are distinguished by the provided number, e.g., #1.

In addition to the integral linear dependency, the variation of the pixel gain for the sensor is also relevant. This variation is derived from a flat-field image. To obtain the flat-field image, the lens is adjusted to be out of focus while observing a homogeneously illuminated white surface. The acquired image is divided by the result of filtering the acquired image multiple times, i.e., after filtering it three times with a median filter (kernel size: 11×11) and three times with a Gaussian filter (kernel size: 11×11). Through spatial filtering of the image, the pixel-wise variations resulting from pixel gain variations are reduced and the illumination field is extracted. The relative frequency of the normalized intensity is shown in Figure 8 for the cameras. The distribution is similar to the expected Gaussian distribution, except for a discrepancy close to 1, where deviations can be noted both to larger and smaller values. The distribution displays an asymmetry regarding the relative frequency at the upper and lower boundaries of the 2σ-interval. A deviation to larger normalized intensities occurs more frequently. Furthermore, in Figure 8, the 2σ-interval of $I_{ref}/I=\pm$ 2.7% is shown.

### 3.3. Temporal Signal Drift

**Figure 9:**
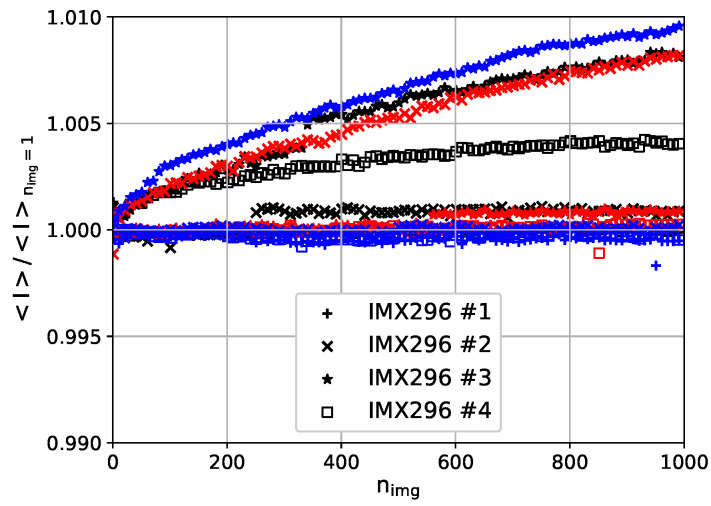
Temporal signal drift of the tested IMX296 cameras. Different brightness levels of the LED are color-coded: 3.0 A (black), 4.5 A (red), 6.0 A (blue). The individual cameras are distinguished by the provided number, e.g., #1.

The measurement sequence, which involves a heating-up phase of several minutes and the combination of multiple camera images into a single result, requires a constant signal over time without arbitrary steps in the intensity signal. Since the CMOS sensor temperature is not controlled, a drift of the measured intensity is to be expected. In a laboratory test, the temporal development is measured by observing a point light source, i.e., a small LED, in 1 m distance. By acquiring the images with four cameras synchronously, the influence of an intensity drift of the light source can be reduced. The development of the spatially averaged intensity normalized by the spatially averaged intensity of the first image <*I*>/<$I_{n_{img}=1}$> is shown in Figure 9 for 1000 consecutive images acquired in approximately 90 s. The test was repeated with the same exposure time but different power settings of the LED. A signal drift of less than 1% variation during the 90 s acquisition time is observed. But the observed drift varies arbitrarily for each measurement, i.e., a dependency on intensity or camera is not observed. In addition to a gradual increase of the deviation <*I*>/<$I_{n_{img}=1}$>, jumps are observed, e.g., at $n_{img}=250$ for camera 2 and the lowest power setting (black), and at $n_{img}\approx 550$ for camera 2 and the medium power setting (red).

### 3.4. TSP Calibration

**Figure 10:**
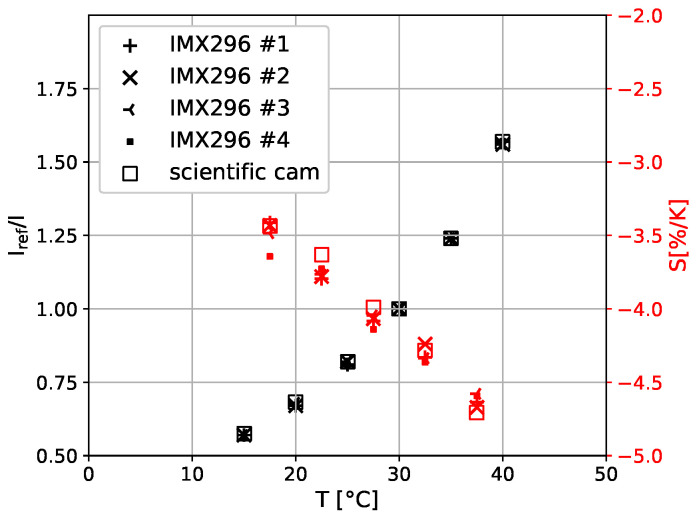
TSP calibration with the four miniature cameras IMX296 in comparison to a monochromatic scientific camera. The Arrhenius diagram $I_{ref}/I$ is provided in black, whereas the temperature sensitivity *S* is red. The reference temperature is $T_{ref}=$30 °C. The individual cameras are distinguished by the provided number, e.g., #1.

The final laboratory test is the calibration of the Europium-based TSP [29] applied to the DLR *SPECTRA-A* flat plate. The calibration is performed in the DLR calibration chamber for the temperature range typical for the application of this TSP coating. The intensity of an averaged area of 51×51 px^2^ is determined on a small TSP sample, which was coated alongside the *SPECTRA-A* flat plate. For values between 15 °C and 40 °C in steps of 5 °C at a total pressure of 100 kPa, the intensity is monitored with all four IMX296 mini cameras and additionally with a monochromatic scientific camera for reference. For each temperature, ten images were acquired for averaging to enhance the signal-to-noise ratio. Additionally acquired dark images for each temperature without LED illumination are subtracted to account for any ambient light and dark currents of the cameras. The normalized intensities $I_{ref}/I$ are displayed in Figure 10, showing good agreement between the individual cameras. Also, the temperature sensitivity *S* is provided in Figure 10. Please note that only the TSP sample was temperature-controlled. The cameras were operated with a constant ambient temperature outside the calibration chamber.

## 4. Visualization of Laminar–Turbulent Transition

Hereafter, flow visualization results from the application of the miniaturized and integrated TSP acquisition system are presented in order to demonstrate the capabilities of the system for an exemplary aerodynamic application. The flow visualizations are used to measure the transition location and the spanwise wavelength of the stationary cross-flow vortices.

**Figure 11:**
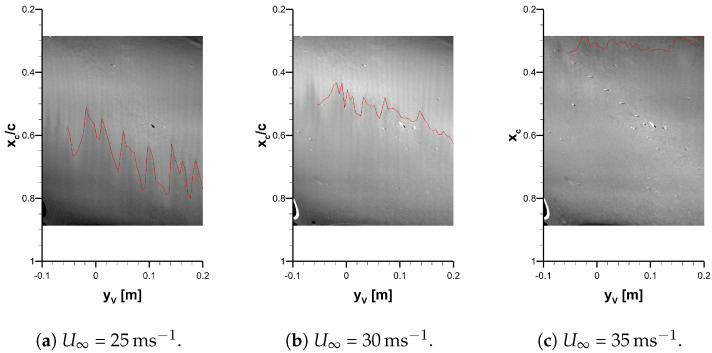
TSP results for the configuration of three DRE layers. The transition location is indicated with a red line.

A subset of three TSP visualizations and the determined transition locations are presented in Figure 11. The results are presented in the vortex-oriented coordinate system and the FOV is limited to the area where the transition location can be evaluated on the entire extent in the flow direction of the cntTSP. These results were acquired in cases using an artificial excitation with three DRE layers (the strongest investigated excitation) at three different velocities, i.e., 25 m s^−1^, 30 m s^−1^, and 35 m s^−1^. The overall impression of the flow visualization changes drastically from the lowest to the largest velocity.

The TSP visualization of the lowest velocity (see Figure 11a) is laminar, up to $x_{c}/c\approx 0.6$, where the typical saw-tooth pattern of the stationary cross-flow instability is observed. The transition location is detected at the location of the saw-tooth pattern. The light gray coloring indicates lower local skin friction associated with laminar flow. The dark color downstream of the saw-tooth pattern indicates the turbulent flow. Within the laminar flow region, the footprints of the stationary cross-flow vortices are visible as dark, parallel lines leading up to the laminar–turbulent transition. Additionally, a variation of the gray level from vortex to vortex is visible. This is most likely caused by a variation in the strength of the cross-flow vortices. This conclusion is supported by the observation that particularly dark vortex footprints can, at many spanwise locations, be traced to particular upstream locations of the laminar–turbulent transition. The footprints of some stationary cross-flow vortices continue seamlessly downstream of the determined laminar–turbulent transition location. A similar observation is reported for the surface-based flow visualization technique naphthalene [33].

The vortex footprints close to the leading edge of the visualization area are hardly visible. At the upstream edge of the visualization area ($x_{c}/c=0.26$), only weak amplitudes of cross-flow vortices are expected. In addition, large-scale variations of the intensity ratio hinder their visualization. In particular, the leading edge of the visualization area is darker when compared to other laminar regions in Figure 11a. The spanwise wavelength of the visualized cross-flow vortex footprints is analyzed via Fourier analysis at $x_{c}/c=0.45$. The deviation of the measured wavelength of $\lambda_{TSP}=$ 9.3 mm from the spanwise spacing of the DRE of λ= 9.5 mm is less than 3%.

When the flow speed is increased to 30 m s^−1^ (see Figure 11b), the laminar–turbulent transition moves upstream, and the extent of the saw-tooth pattern in the flow direction is reduced. When increasing the flow speed to 35 m s^−1^ (see Figure 11c), the laminar–turbulent transition is located at the upstream end of the visualization area, i.e., close to the location of the backward-facing step. The saw-tooth pattern mostly disappears. This observation in flow visualization techniques could be caused by the increasing significance of the traveling cross-flow instability in the laminar–turbulent transition process [34].

**Figure 12:**
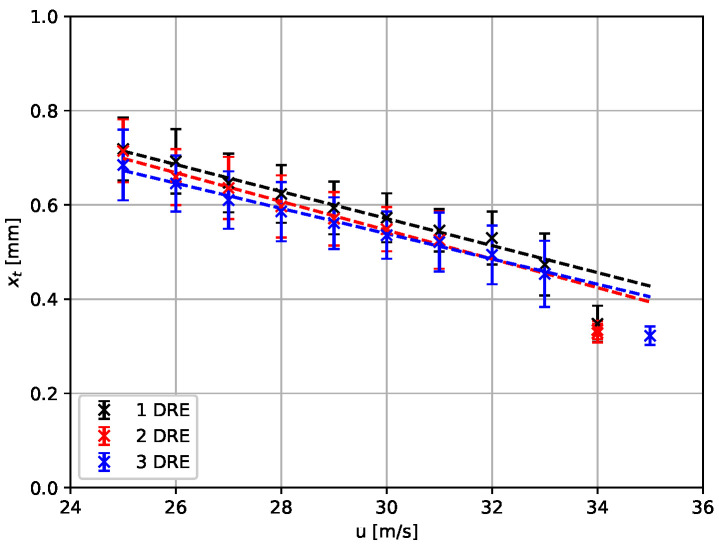
Average transition location and standard deviation for a variation of the flow velocity for three different DRE heights. The dashed lines indicate the linear trend of the development of the transition.

The transition location in the velocity range of 25 m s^−1^ to 35 m s^−1^ and for one, two, and three layers of the DRE with a backward-facing step at $x_{c}/c=0.45$ is presented in Figure 12. For the *SPECTRA-A* experiment, a gradual reduction of the extent of laminar flow can be observed up to $U_{\infty }$ = 33 m s^−1^. All three configurations have a similar gradual reduction with an average difference between the transition location with one and three layers of DRE of $\Delta x_{c}/c\approx 0.03$. In addition, the streamwise extent of the saw-tooth pattern is evaluated by computing the standard deviation of the spanwise distribution of the transition location. A comparison of the results shown in Figure 11 shows that the saw-tooth pattern is pronounced most at the lowest velocity. With increasing velocity, the streamwise extent decreases. The standard deviation of the transition location does not significantly vary between the investigated configurations. Between $U_{\infty }$ = 33 m s^−1^ and 34 m s^−1^, a critical effect of the backward-facing step on the transition location can be observed. The transition location moves further upstream compared to the development at lower velocities. This is clearly indicated by the deviation from the dashed lines in Figure 11, which provide the linear trend of the movement of the transition location in the velocity range of 25 m s^−1^ to 33 m s^−1^. For details on the effects of backward-facing steps on the cross-flow-dominated laminar–turbulent transition, see the works of Eppink, Duncan, and Tufts [19,20,35].

Observations on sometimes subtle differences of the spanwise average as well as the spanwise variance of the transition location can enable detailed interpretations in aerodynamic applications, as in this case for research on cross-flow-dominated transition. Such interpretation is outside the scope of this publication; however, the presented exemplary results show that the inexpensive setup described in this publication is able to visualize the laminar–turbulent transition in a large area with sufficient resolution and accuracy, and is suitable for transition research in swept configurations.

## 5. Discussion

From the performed laboratory tests, the characteristics of the camera are evaluated with respect to its suitability for TSP measurements. This ideally includes a linear dependency between the incidence light and the output signal, a homogeneous sensitivity of the pixels, and a steady signal for constant light incidence.

First, a linear dependency between the incidence light and the output signal is very important for the TSP measurement technique because of the division during the data evaluation. The tested cameras show linear behavior for the range of tested exposure times. This linear behavior is also valid when close to saturation. Thus, the linearity characteristics of the camera are suitable for the TSP measurement technique.

Second, the investigation of the sensitivity homogeneity of the pixels showed that the 2σ-interval corresponds to $I_{ref}/I=\pm 2.7$. For the TSP [29] applied to the wind tunnel test, the 2σ-interval corresponds to a temperature variation of approximately ±0.8 K at $T_{ref}=$ 30 °C. When the cameras are applied for TSP visualizations, this error will be observed as image noise and could require increased heating power to increase the temperature difference between the laminar and turbulent boundary layers.

Third, the temporal stability of the acquired signal, when a constant light source is observed, showed a signal drift of less than 1% variation during the 90s acquisition time. But the observed drift varied arbitrarily for each measurement, i.e., a dependency on the intensity or camera is not observed. Furthermore, arbitrary jumps are observed in the signal. These observations of the camera behaviors could not be fully explained by temperature effects because the cameras were operated in a laboratory with air conditioning and the cameras were very close to each other. Also, the data acquisition was performed simultaneously for all cameras. A variation of less than 1% corresponds to a temperature variation of $\Delta T_{error}\approx$ 0.3 K at $T_{ref}=$ 30 °C, which is also acceptable for TSP visualizations.

The calibration of the TSP sample provides the possibility of directly comparing the IMX296 mini cameras with a scientific camera. In the Arrhenius diagram in Figure 10, a good agreement between the intensity ratio for the IMX296 mini cameras and the scientific camera is observed. The temperature sensitivity derived from the intensity measurement with the IMX296 mini cameras has an average deviation of ΔS≈ 0.05% K^−1^, which provides an acceptable agreement. But the scatter of the temperature sensitivity measured by the miniature cameras shows larger variation and random deviations of up to ΔS≈ 0.13% K^−1^. These deviations occur despite using a large number of pixels for averaging the intensities during calibration.

These observed deviations of the determined temperature sensitivity in combination with the low temperature resolution for a single image (see Table 1), as well as the uncertainties caused by the insufficient homogeneity of the pixel sensitivity and the temporal behaviors of the IMX296 mini cameras are not deemed suitable for quantitative temperature measurements with the TSP method. Nevertheless, the IMX296 mini camera is deemed suitable for conducting TSP visualizations.

The TSP results presented in Figure 11 prove that the installation of the IMX296 mini cameras in the *SPECTRA-A* configuration is successful. The optical setup developed in laboratory pre-tests resulted in a FOV of $\delta x_{c}\times \delta y_{c}\approx$ 350 mm × 550 mm with an image resolution of 0.2 mm/px to 0.4 mm/px. This setup is capable of resolving small changes in the transition location and differences in the footprints of cross-flow vortices. For comparison, a test setup with the *SPECTRA-A* configuration in the 1MG with a PCO.4000 and telephoto lens was conducted. Due to the required position of the camera outside of the open test section, the camera is located 2.5 m downstream of the trailing edge. With the limitations in the 1MG, a spatial resolution of 0.3 mm/px is achieved. Thus, the spatial resolutions of the setup with a scientific camera and an integrated setup with the miniature camera are similar. Furthermore, the required temperature ratios of the TSP surface temperature and the flow temperature $T_{surface}/T_{fluid}$ are compared for measurements with a scientific and miniature camera setup. The necessary temperature ratio for achieving satisfying visualization results can provide an indication of the sensitivity of the measurement setup. In a previous TSP measurement, cross-flow instabilities with scientific camera [31] temperature ratios of up to $T_{surface}/T_{fluid}\approx 1.08$ were investigated. In the current setup with the miniature camera, a lower temperature of $T_{surface}/T_{fluid}\approx 1.01$ was suitable for acquiring the TSP data. When comparing the current results with TSP visualizations acquired with scientific cameras of stationary cross-flow instability-induced transition [31], it is noticeable that the current results are more noisy. Nevertheless, the visualizations presented in this paper clearly demonstrate the suitability of qualitative, time-averaged TSP visualizations. These visualizations are suitable for transition measurements. Furthermore, the footprints of the cross-flow vortices are resolved, which are used to transfer the TSP results from the plate-oriented coordinate system to the vortex-oriented coordinate system.

The quality of the results could be further enhanced by increasing the number of acquired images and increasing the temperature ratio between the TSP surface and the flow. Also, reference images are acquired for each velocity to avoid issues with the pixel-gain error, which can be emphasized by the mapping process when compensating for model deformation. Also, reference images should be acquired at every tested velocity to minimize the effects of the model deformation.

The integration of reliable LEDs for TSP excitation proved successful, despite the assessment that the distribution of the excitation light should be improved to a more uniform distribution. Due to the fixed installation in the displacement body, the current setup lacks the possibility of adjusting the lighting. Hence, the method for designing the lighting must be revised.

The development allows enhancing the range of TSP applications to a permanently installed optical measurement system, e.g., model integration, or applications where it is likely to damage the optical measurement system during the measurements. One advantage specific to the *SPECTRA-A* configuration with the integrated TSP setup is the possibility of working with the same setup in a different wind tunnel. The integrated optical measurement system provides results that are useful for an aerodynamic analysis at a low budget, i.e., approximately one-tenth of a standard scientific setup. Furthermore, the low price of the acquisition system of under EUR 1000 per camera will increase the number of potential TSP users. The system per camera includes a camera, a single-board computer to control the camera, a lens that includes an appropriate optical filter, and a high-power LED to excite the TSP.

## 6. Conclusions

In this paper, the efficacy of the machine-vision camera, VC MIPI IMX296, for flow visualizations using the TSP method is validated through laboratory investigations and transition measurements on the *SPECTRA-A* configuration:

- Laboratory investigations revealed that the camera offers commendable linearity and a tolerable temporal drift. However, unpredictable fluctuations in the intensity signal and inconsistent behaviors across different cameras and measurements were observed.
- The integration of the cameras and LEDs into the *SPECTRA-A* configuration could be achieved easily due to the small dimensions.
- The achieved TSP visualization quality is good because small changes of the transition location and differences in the footprints of cross-flow vortices are resolved.
- The low price of under EUR 1000 for each camera, including all accessories, provides an inexpensive optical acquisition system for TSP measurements, thereby reducing the initial system costs for new groups of TSP-users.

## Figures and Tables

**Table 1:** Comparison of characteristics of IMX296 with a scientific monochromatic camera. ${\left(\Delta T\right)}_{min}$ is calculated with the procedure described by [7], using the characteristics of TSP integrated into the *SPECTRA-A* configuration.

	IMX296	PCO.4000
resolution [px]	1440×1080	4008×2672
dynamic [bit]	10	14
full-well capacity [$e^{-l}$]	11,000	60,000
w×h×l [mm]	22×23.5×10	65×85×211
${\left(\Delta T\right)}_{min}$ [K]	0.42	0.18

## Data Availability

Data is available upon reasonable request.
